# 

**Published:** 1894-09-15

**Authors:** 


### CONTENTS.

**Vaccination and Compulsory Vaccination** - - - 475

**Around the Hospitals** - - - - - - - - 476

**The Psychologist in History**—Madness or —— What? III.—The Kings of Spain - - - - - - - - 477

**Modern Sociology.**—Labour and Health - - - - 478

**A Bureau of Autopsy** - - - - - - - - 478

**Annotations.**—The Golf "Cure" for Insomnia—A Clinal Research Association—School Diphtheria in Towns - - - 479

**Notes and News** - - - - - - - - 480

**Scraps and Gleanings** - - - - - - - 490

**Small-pox and Vaccination at Leicester** - - - 491

**The Student of Medicine** - - - - - - 491

**Medical Progress and Hospital Clinics:**

Chronic Catarrh of the Middle Ear —IV. - - - - 481

PROGRESS IN MEDICINE AND SURGERY.—Diseases of the Heart and Vascular System. II. - - - -

Surgery of the Lungs and Pleura - - - - - 483

THE BRITISH MEDICAL ASSOCIATION AT BRISTOL - - - 484

NEW APPLIANCES AND THINGS MEDICAL - - - - 485

**The Institutional Workshop:** - - - - - 486

THE ROYAL VISIT TO BIRMINGHAM

THE STORY OF THE INSANE FROM YEAR TO YEAR - - 487

PRACTICAL DEPARTMENTS.—The Hospital Laundry - - 489

CONSTRUCTION NOTES - - - - - - - 489

490

### EXTRA SUPPLEMENT.—THE NURSING MIRROR.

**News from the Nursing World.**—Royal Visitors at Birmingham—Easy and Efficient Disinfection—Out of Town—Probationers' Duties—The Royal British Nurses' Association—Ashford Cottage Hospital—The Paupers' Saucepan—English Nurses in Hong Kong — Nurses at Coblentz — Spaniards at the Sea—State Registration—Short Items - - ccxxxiii

**Diet in Disease.**—By Mrs. ERNEST HART. II.—Scurvy and Scrofula - - - - - - - - - - - ccxxxv

**Nursing in Germany** - - - - - - - ccxxxvi
**A Home for the Dying** - - - - - - ccxxxvi
**A Case for Assistance** - - - - - - ccxxxvi
**Nursing on the Labrador** - - - - - ccxxxvii
**Everybody's Opinion** - - - - - - ccxxxviii
**Nursing in South Africa** - - - - - ccxxxviii
**Notes and Queries** - - - - - - - ccxxxviii
**Wants and Workers** - - - - - - - ccxxxviii

